# Five Different Piscidins from Nile Tilapia, *Oreochromis niloticus*: Analysis of Their Expressions and Biological Functions

**DOI:** 10.1371/journal.pone.0050263

**Published:** 2012-11-30

**Authors:** Kuan-Chieh Peng, Shu-Hua Lee, Ai-Ling Hour, Chieh-Yu Pan, Lin-Han Lee, Jyh-Yih Chen

**Affiliations:** 1 Marine Research Station, Institute of Cellular and Organismic Biology, Academia Sinica, Ilan, Taiwan; 2 Department of Life Science, Fu-Jen Catholic University, Taipei, Taiwan; Bioinformatics Institute, Singapore

## Abstract

Piscidins are antimicrobial peptides (AMPs) that play important roles in helping fish resist pathogenic infections. Through comparisons of tilapia EST clones, the coding sequences of five piscidin-like AMPs (named TP1∼5) of Nile tilapia, *Oreochromis niloticus*, were determined. The complete piscidin coding sequences of TP1, -2, -3, -4, and -5 were respectively composed of 207, 234, 231, 270, and 195 bases, and each contained a translated region of 68, 77, 76, 89, and 64 amino acids. The tissue-specific, *Vibrio vulnificus* stimulation-specific, and *Streptococcus agalactiae* stimulation-specific expressions of TP2, -3, and -4 mRNA were determined by a comparative RT-PCR. Results of the tissue distribution analysis revealed high expression levels of TP2 mRNA in the skin, head kidneys, liver, and spleen. To study bacterial stimulation, *S. agalactiae* (SA47) was injected, and the TP4 transcript was upregulated by >13-fold (compared to the wild-type (WT) control, without injection) and was 60-fold upregulated (compared to the WT control, without injection) 24 h after the *S. agalactiae* (SA47) injection in the spleen and gills. Synthesized TP3 and TP4 peptides showed antimicrobial activities against several bacteria in this study, while the synthesized TP1, -2, and -5 peptides did not. The synthesized TP2, -3, and -4 peptides showed hemolytic activities and synthesized TP3 and TP4 peptides inhibited tilapia ovary cell proliferation with a dose-dependent effect. In summary, the amphiphilic α-helical cationic peptides of TP3 and TP4 may represent novel and potential antimicrobial agents for further peptide drug development.

## Introduction

Antimicrobial peptides (AMPs) are cationic peptides that play important roles in innate immunity. Fish live in pathogen-rich aquatic environments and depend on their innate immune systems to resist pathogen infections; thus, they produce novel secretions of antimicrobial materials such as AMPs [1]. In recent years, a growing body of research has revealed the forceful antimicrobial and antiendotoxin activities of AMPs [2]–[4]. AMPs are positively charged amphipathic molecules characterized from a wide variety of plants and animals (including fish and shrimp) that act as a natural defense mechanism [5]–[7]. AMPs are membrane-active poly-cationic peptides with an affinity to destroy bacterial membranes by forming pores with barrel-stave, carpet, or toroidal pore mechanisms, and their negatively charged microbial surfaces rely on electrostatic interactions and biochemical structures [8]–[11].

Piscidins are cationic AMPs expressed by fish mast cells [12]. The piscidin family consists of structurally related mature peptides of 21∼44 residues that possess an amphipathic α-helical structure, which suggests that piscidins have great bactericidal activities against a variety of microorganisms [13], [14]. The piscidin family includes pleurocidin, moronecidin, chrysophsin, dicentracin, epinecidin-1, and myxinidin. Piscidins have a widespread presence in higher teleosts, so far including 11 species in eight families of the Moronidae, Sciaenidae, Sparidae, Latidae, Siganidae, Belontidae, Cichlidae, and Perichthyidae [15]–[17]. The literature provides indirect evidence which implies that piscidins may have roles in antimicrobial defense systems in fish innate immunology. For example, synthetic piscidin 2 from hybrid striped bass was active *in vitro* against *Candida albicans* (ATCC 90028), *Malassezia furfur*, and *Trichosporon beigelii* at respective minimal inhibitory concentrations (MICs) of 6.25, 6.25, and 1.56 µM [18]. The synthetic piscidin 1 (peptide sequence: FFHHIFRGIVHVGKTIHRLVTG) showed an MIC of 3.1 µM against methicillin-resistant *Staphylococcus aureus* (MRSA) in an *in vitro* assay [19]. The synthetic piscidin 2 presented potent activity against the water mould *Saprolegnia*, and the minimum oomyceticidal concentration (MOC 100) ranged 12.5∼25 µg/ml [20]. Those results suggest that piscidins are similar to other AMPs which also possess antimicrobial activities [21]. Fish piscidins possess antimicrobial activities *in vitro*, and in fish, are stored in granules of phagocytic granulocytes and are delivered to pathogen-containing phagosomes upon phagocytosis [22], which suggests that fish may use piscidins as antimicrobial agents *in vivo* to kill invading pathogens. Another interesting research result suggested that positively selected sites identified in the Atlantic cod (*Gadus morhua* L.) piscidin gene that codes for the mature peptide are associated with the adaptation of piscidins to pathogens and may be involved in protecting the host against rapidly evolving pathogens [23]. The Atlantic cod paralogues were termed gaduscidins (GAD-1 and GAD-2). These GAD transcripts are highly constitutively expressed in immune-related organs such as the spleen and head kidneys and are weakly induced in the spleen following an intraperitoneal (IP) injection of bacterial antigens [24]. Recently, two piscidin paralogues (pis1 and pis2) and a novel splice variant (pis2-β) in Atlantic cod were reported. Synthetic pis2 and pis2-β had great antiparasitic activity against *Tetrahymena pyriformis* but had limited or no antibacterial activity, respectively. These research results mentioned above support cod piscidins being important immune-related genes in the cod innate immune system and playing multifunctional roles in Atlantic cod following their structural diversification [25].

Tilapia is a cultured teleost that is highly economically important in China, Taiwan, and other Asian countries. Unfortunately, bacterial diseases are major limiting factors in tilapia culture, and several bacterial pathogens have caused major problems for tilapia aquaculture [26]. Therefore, it is necessary to find and investigate new AMPs in tilapia such as piscidins, and apply tilapia piscidins to produce new vaccines or antimicrobials as feed supplements for fish culturists and aquaculture in general as therapeutic tools against bacterial infections. As a first step in our research, we determined Nile tilapia (*Oreochromis niloticus*) piscidins by examining a tilapia EST database and isolated five piscidin-like complementary (c)DNA clones from the Nile tilapia spleen. They were named Nile tilapia piscidin 1 (TP1), -2, -3, -4, and -5. In the second step, we determined the messenger (m)RNA expression patterns and bactericidal activities of synthetic Nile tilapia piscidins against gram-negative and -positive bacteria. In the third step, we determined whether Nile tilapia piscidins inhibited cell viability and hemolytic activity. Taken together, our results suggest that each of these five Nile tilapia piscidins has specific functions and may provide new peptide drugs for development for both aquaculture and biopharmaceutics.

## Materials and Methods

### Ethics statement

All animal handing procedures were in accordance with Academia Sinica guidelines. All procedures were approved by the Animal Care and Use Committee of Academia Sinica.

### Bacterial strains and culture conditions

The gram-negative bacteria, *Aeromonas hydrophila* (BCRC 13018), *Pseudomonas aeruginosa* (ATCC 19660), *Vibrio alginolyticus* (from Dr. Chun-Yao Chen, Tzu Chi University, Hualien, Taiwan), and *V. vulnificus* (204; from Dr. Chun-Yao Chen); and the gram-positive bacteria, *Enterococcus faecalis* (BCRC 10066), *Streptococcus agalactiae* (BCRC 10787), and *S. agalactiae* (819; from Dr. Chun-Yao Chen) were cultured in media and at temperatures suggested by the Food Industry Research and Development Institute (BCRC; Hsinchu, Taiwan; http://www.bcrc.firdi.org.tw:819/bcrc/indexe.htm) or American Type Culture Collection (ATCC, Manassas, VA, USA; http://www.atcc.org/Home.cfm). Other bacteria from Dr. Chun-Yao Chen were cultured in trypticase soy broth and agar at 37°C following his suggestions. *Escherichia coli* for transformation was cultured in LB medium at 37°C.

### Isolation of Nile tilapia piscidin cDNA clones

*Oreochromis niloticus* spleen tissues were collected for RNA extraction following a previous publication [27]. The Nile tilapia piscidin coding region was amplified by a polymerase chain reaction (PCR) using GR647653.1 (named TP1), GR604642.1 (XM_003456635; named TP2), GR645750.1 (XM_003456614; named TP3), GR634176.1 (XM_003456613; named TP4), and GR648328.1 (named TP5) from PubMed GenBank data. The PCR was carried out in a final volume of 50 µl. The reaction consisted of 2 µl of *O. niloticus* first-strand cDNA from spleen mRNA as the PCR template, 5 µl of 10× PCR buffer, 200 µM of each of the dNTPs, 2.5 units of Prime Taq™ DNA Polymerase (GENET BIO, Chungnam, Korea), and 0.2 µM of the following primers: TP1 5′ (5′-ATGAAGTCTGCTGTGATCTTTCTTGTGC) and TP1 3′ (5′-CTAGTCAAATTCCCGTTGACGCA); TP2 5′ (5′-ATGAAGTGTGCTGCAGTATTTCTTATGCTGTCC) and TP2 3′ (5′-CTAGTCAAAATTAAGTCGACGAGGGT); TP3 5′ (5′-ATGAAGTGCACCATGCTGTTCCTTGTGCTGTCGATGGTT) and TP3 3′ (5′-CTAGTTAAAAGCAGCCCTTTCCC); TP4 5′ (5′-ATGAAGTGCACTATACTGTTCCTTGTGCTGTCGATGGTG) and TP4 3′ (5′-CTAGTTAAAAGCAACTCTCTCTCGTTTG); and TP5 5′ (5′-ATGAAGTCTGCCATAATCTTTCTTGTAT) and TP5 3′ (5′-CTATGACATCACAGCATCTTCAAATTC). The reaction mixture was subjected to 35 PCR cycles of 20 s at 94°C (denaturation), 20 s at 55°C (annealing), and 20 s at 72°C (extension); the PCR products were then resolved on 2% agarose gels. The PCR products were cloned into a pCR-Blunt (Invitrogen, CA, USA) and transformed into the DH5α *E. coli* strain, and then 10 recombinant clones were chosen to sequence and identify the tilapia piscidins.

### Sequence analysis and construction of Nile tilapia piscidin structural models

Structural models of the five types of Nile tilapia piscidin were respectively constructed using the crystal structure of piscidin 4 as a template [28]. The amino acid sequences of Nile tilapia piscidins were obtained from the cloned cDNA of this study. A Schiffer-Edmundson representation of Nile tilapia piscidin was obtained with the Helical Wheel Projections software available online (http://rzlab.ucr.edu/scripts/wheel/wheel.cgi). Nile tilapia piscidin amino acids and the other piscidins were input into the website, http://psort.hgc.jp/form2.html, for further analysis.

### Quantification of mRNA levels by comparative reverse-transcription (RT)-PCR

Nile tilapia (*O. niloticus*) were kept at the Marine Research Station, Institute of Cellular and Organismic Biology, Academia Sinica. Nile tilapia were kept in fiberglass-reinforced plastic tanks with a 12-h dark∶ 12-h light photoperiod. The temperature of the water was maintained at 25°C. The fish were fed daily *ad libitum* with a commercial diet (Uni-President Group, Taiwan). Fish were humanely killed by immersion in an anesthetic bath containing of 3-aminobenzoic acid ethyl ester (0.6 g·L^−1^, Sigma). Six Nile tilapia (*O. niloticus*) fish tissues were collected for extraction of RNA following a previous publication [27]. For tissue distribution studies, tissues of the skin, gills, head kidneys, liver, spleen, and intestines were collected for total RNA preparation following the same method described above. Levels of Nile tilapia piscidin mRNA were determined following a comparative real-time RT-PCR analysis of the tissue distribution after *V. vulnificus* (204) stimulation (5×10^5^ colony-forming units (cfu)/fish, with an average fish body weight of 12 g), or *S. agalactiae* (SA47) stimulation (5×10^6^ cfu/fish). Pathogens were intraperitoneally injected. The mRNA was transcribed to first-strand cDNA, and single-stranded cDNA was used in the real-time comparative quantitative PCR for detecting relative expression levels of piscidin and ubiquitin (XM_003449030). The PCR followed our previous publication [29]. Primers for the TP2 gene were the forward 5′-primer (AGCCAAACATTTTCTTCATCG) and reverse 3′-primer (AACGAAAACAACCTGTAGGAA); for the TP3 gene were the forward 5′-primer (GGGAGGCCTTTATTCACCAT) and reverse 3′-primer (TGCTGCTGTTGTTGCTGTTT); for the TP4 gene were the forward 5′-primer (GGTCGTCCTCATGGCTGA) and reverse 3′-primer (GTCGTATGAGGCGATGGATAG); and for the Nile tilapia ubiquitin gene were the forward 5′-primer (TGAGCCCAGTGACACTATTGA) and reverse 3′-primer (TTTGCCAGCAAAGATCAGAC). Primers were designed using the Roche online program Universal ProbeLibrary Assay Design Center (http://www.roche-applied-science.com/sis/rtpcr/upl/index.jsp?id=UP030000). All samples were examined in triplicate, and the experimental process was the same as that in a previous publication [29]. Values are presented as the mean (± standard error; SE). Finally, to evaluate if there was any genomic contamination, a ubiquitin (XM 003449030) primer set amplifying a region including an intron was used in our study. All quantitative (q)PCR products of the target and control genes were sequenced, and the results confirmed their identity. No-template negative controls were included in all of our experiments, and the results showed that no amplification was detected. The significance was calculated and determined by Student's *t*-tests using the SPSS statistical program (SPSS 15.0, Chicago, IL, USA), and significance was set to (*) *p*<0.05. Comparative real-time RT-PCR products of the target and control genes were sequenced, and the results confirmed their identity. The amplification efficiency of each primer set was assessed using cDNAs from Nile tilapia serially diluted 2-fold (Supplementary Table 1, Supplementary Figs. 1, 2). Expression levels of piscidin genes relative to that of ubiquitin were calculated by the comparative Ct method [30].

### Synthesis of the five Nile tilapia piscidin peptides and bacteriostatic analysis

Peptides were synthesized by GL Biochem (Shanghai, China) using a solid-phase procedure of Fmoc chemistry. Crude peptides were extracted, lyophilized, and purified by reverse-phase high-performance liquid chromatography (HPLC). The molecular masses and purities of the purified peptides were respectively verified by mass spectroscopy and HPLC. Synthetic peptides at >95% purity were reconstituted in phosphate-buffered saline (PBS; pH 7.4) for the experiments. The TP1 sequence was FDWDSVLKGVEGFVRGYF, the TP2 sequence was GECIWDAIFHGAKHFLHRLVNP, the TP3 sequence was FIHHIIGGLFSVGKHIHSLIHGH, the TP4 sequence was FIHHIIGGLFSAGKAIHRLIRRRRR, the TP5 sequence was QLQGKQVSGEVVQKVLQELIQSVAKP, the epinecidin-1 (Epi-1) sequence was GFIFHIIKGLFHAGKMIHGLV, and the cod pis 1 sequence was FIHHIIGWISHGVRAIHRAIHG. C terminals of all synthetic peptides were amidated. The minimal inhibitory concentration (MIC) was measured for each strain of microorganism listed above. The MICs of the peptides were determined by a broth microdilution analysis based on an online method (http://cmdr.ubc.ca/bobh/methods/MODIFIEDMIC.html; Hancock Laboratory Methods. Department of Microbiology and Immunology, University of British Columbia, British Columbia, Canada) without modification.

### In vitro cytotoxicity assay

The in vitro assays used tilapia ovary (TO2) cells [31]. After 24 h of incubation, cells were treated with peptides (0∼100 µg/ml) and incubated for another 24 h. Cell viability was measured at the end of treatment by the addition of 20 µl of the tetrazolium compound, 3-(4,5-dimethylthiazol-2-yl)-5-(3-carboxymethoxyphenyl)-2-(4-sulfophenyl)-2H-tetrazolium inner salt (MTS), following our previous publication without modification [32]. Experiments were repeated three times. Results are expressed as a percentage of the survival rate of viable cells.

### Hemolytic assay

The hemolytic activities of Nile tilapia piscidins were determined according to a previous method [33], [34]. In brief, erythrocytes of tilapia (2×10^7^ cells) were washed three times with 0.9% NaCl and incubated with various concentrations of the peptides for 3 h at 37°C. After centrifugation, the absorbance at 545 nm of the suspension was determined. Erythrocytes in PBS (A*_blank_*) and 0.1% Triton X-100 (A*_triton_*) respectively served as the negative and positive controls. The percentage of hemolysis was calculated according to the equation described in a previous publication [25]. The hemolysis assay was performed in triplicate.

## Results

### Characterization of five Nile tilapia piscidin cDNA sequences

**Figure 1:**
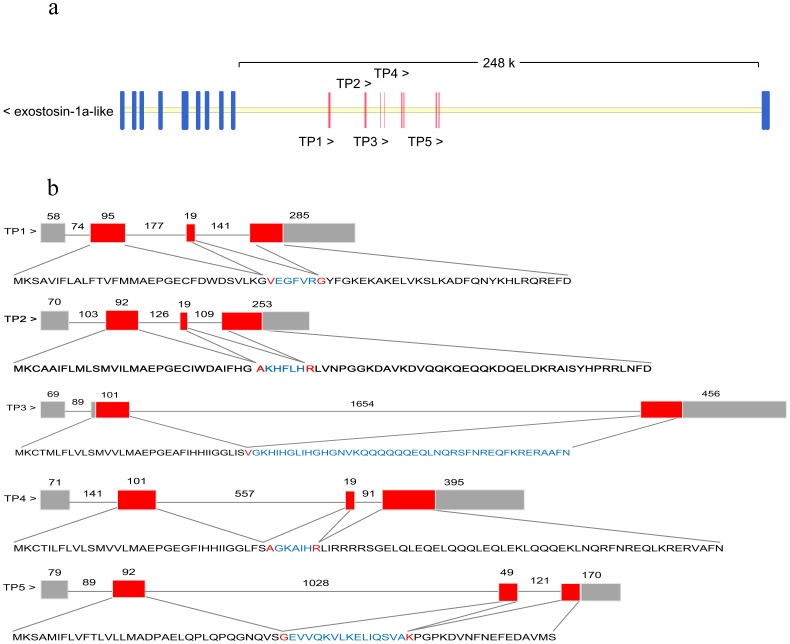
Genomic structure and location of Nile tilapia piscidin genes. a. Comparison of the genomic organization of piscidin genes of Nile tilapia. The five Nile tilapia piscidin genes were located between the ninth and tenth exons of exostosin-1a-like. Both were transcribed in reverse. b. The length of the coding sequence of each exon is indicated by numbers above the boxes, and each intron is indicated by numbers above the line. Red indicates regions of translated amino acid sequences, and gray indicates the 5′ and 3′ untranslated regions (UTRs).

cDNA coding regions of five different piscidin sequences (Supplementary Fig. 3) were isolated and characterized. The five cDNA sequences were named TP1∼5, the cDNAs of which respectively encoded 68, 77, 76, 89, and 64 amino acids. A potential cleavage site for the signal peptide was predicted to be between Pro19 and Gly20 for TP1, between Pro19 and Gly20 for TP2, between Pro19 and Gly20 for TP3, between Ala17 and Glu18 for TP4, and between Leu22 and Gln23 for TP5. In addition, we found one consensus nuclear localization sequence (NLS) in the Nile tilapia piscidin TP4 amino acid sequence, located at amino acid number 43 (RRRR). The mature peptide cleavage site was analyzed by PSORT II Prediction (http://psort.hgc.jp/form2.html), and the potential cleavage site of the signal peptide was predicted. Comparison with the genomic sequence published on the website of the NCBI Reference Sequence: NT_167592.1 showed that TP1∼5 were located on the same chromosome (Fig. 1a), and were spread in the exostosin-1a-like gene intron region. The Nile tilapia piscidin gene and exostosin-1a-like gene were reverse-transcribed for each gene transcription. TP1, -2, -4, and -5 possessed a four-exon/three-intron structure, while the TP3 gene possessed a three-exon/two-intron structure (Fig. 1b). All introns possessed the classic 5′ GT/AG 3′ donor and acceptor site rule after comparison of cDNA sequences with published genomic DNA sequence information from NCBI.

**Figure 2:**
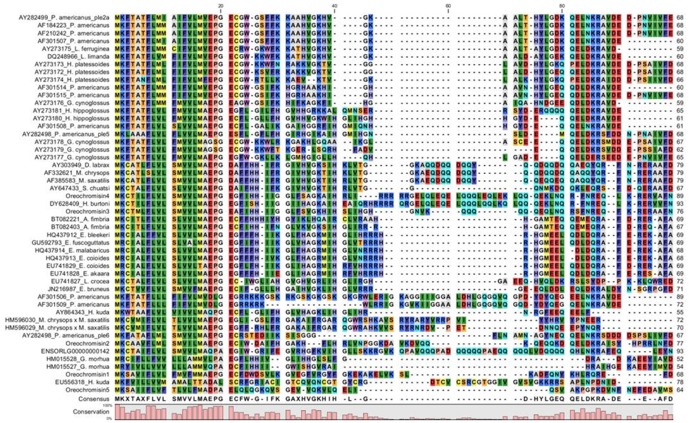
Sequence alignment between piscidins. Multiple sequence alignments of the Nile tilapia piscidin peptides with selected piscidin or with other members of the piscidin family. Gaps were inserted to obtain maximum homology. Sequences used for comparison with the references for GenBank accession numbers are described in Supplementary Table 3. All gene sequences of the coding region were input into the dendogram for alignment. The same amino acid is indicated by the same color, such as methionine (M) being indicated by yellow.

Alignment of proteins from different species showed that piscidin sequence similarities among fish piscidins were remarkably high (Fig. 2). The blast analysis showed that the sequence similarity of TP1 with TP2 was 51%, that of TP1 with TP3 was 40%, that of TP1 with TP4 was 61%, that of TP1 with TP5 was 37%, that of TP2 with TP3 was 48%, that of TP2 with TP4 was 52%, that of TP2 with TP5 was 26%, that of TP3 with TP4 was 63%, that of TP3 with TP5 was 48%, and that of TP4 with TP5 was 52%. These sequences were input for NCBI blast analyses, and results suggested that TP1 was related to the *O. niloticus* pleurocidin-like peptide, WF4-like (accession no.: XM_003456635), with an identity of 43%. TP2 was also related to the same pleurocidin-like peptide, WF4-like (accession no.: XM_003456635), with an identity of 100%. TP3 was related to the *O. niloticus* dicentracin-like peptide (accession no.: XM_003456614) with an identity of 97%. TP4 was related to the *O. niloticus* moronecidin-like peptide (accession no.: XP_003456661) with an identity of 98%. TP5 was related to the *Epinephelus bruneus* piscidin-like peptide (accession no.: JN216987.1) with an identity of 34%.

### Structural model analysis of Nile tilapia piscidins

**Figure 3:**
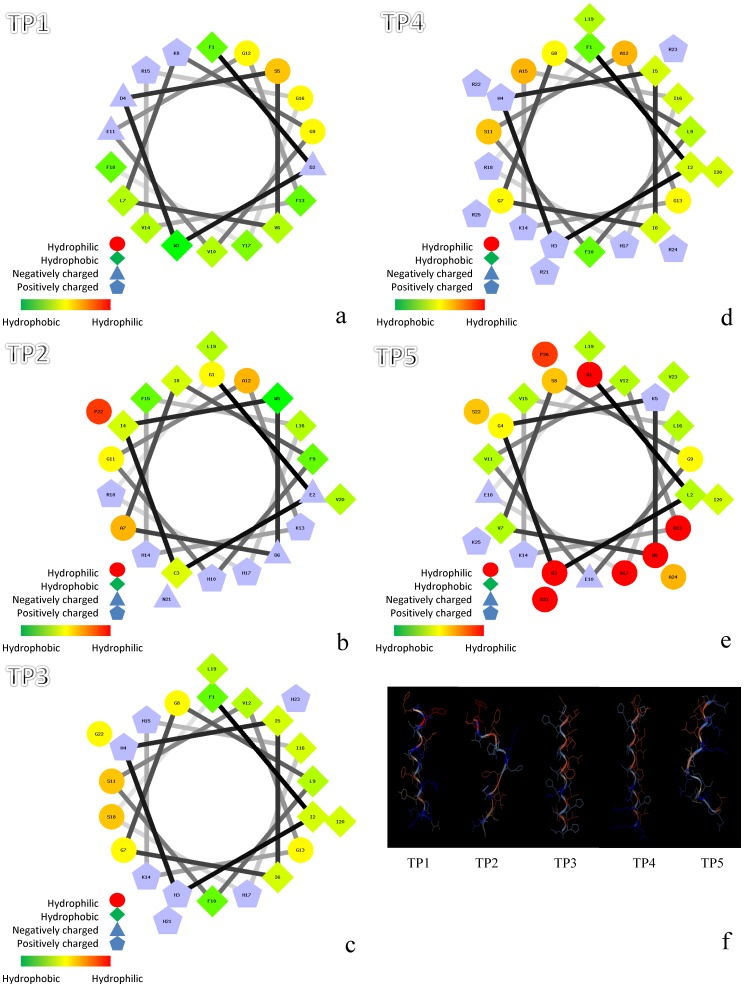
Amphipathic α-helical structures and three-dimensional structures of five different Nile tilapia piscidins. A Schiffer-Edmunson plot of (a) Nile tilapia piscidin 1 (TP1), (b) -2, (c) -3, (d) -4, and (e) -5 was produced with the PEPWHEEL program. Amino acid residues are successively numbered and connect each amino acid following the sequence with lines that represent its relative position along the helix. Red indicates hydrophilic, green indicates hydrophobic, blue triangles indicate negatively charged, and blue pentagons indicate positively charged residues. Structural models of (f) TP1∼5. Pictures were generated with the Viewerlite program (Accelrys, San Diego, CA, USA).

As indicated in Fig. 3a–e, Schiffer-Edmunson helical wheel modeling was used to predict hydrophobic and hydrophilic regions in the five synthesized Nile tilapia piscidins. TP1 showed a hydrophobic slant to one side, while TP5, and to a lesser extent TP2, exhibited limited amphipathicity. The five tilapia piscidin peptides were predicted to form an amphipathic α-helix (Fig. 3f).

### Nile Tilapia piscidin constitutive mRNA expressions and transcript expression responses to bacterial infection

**Figure 4:**
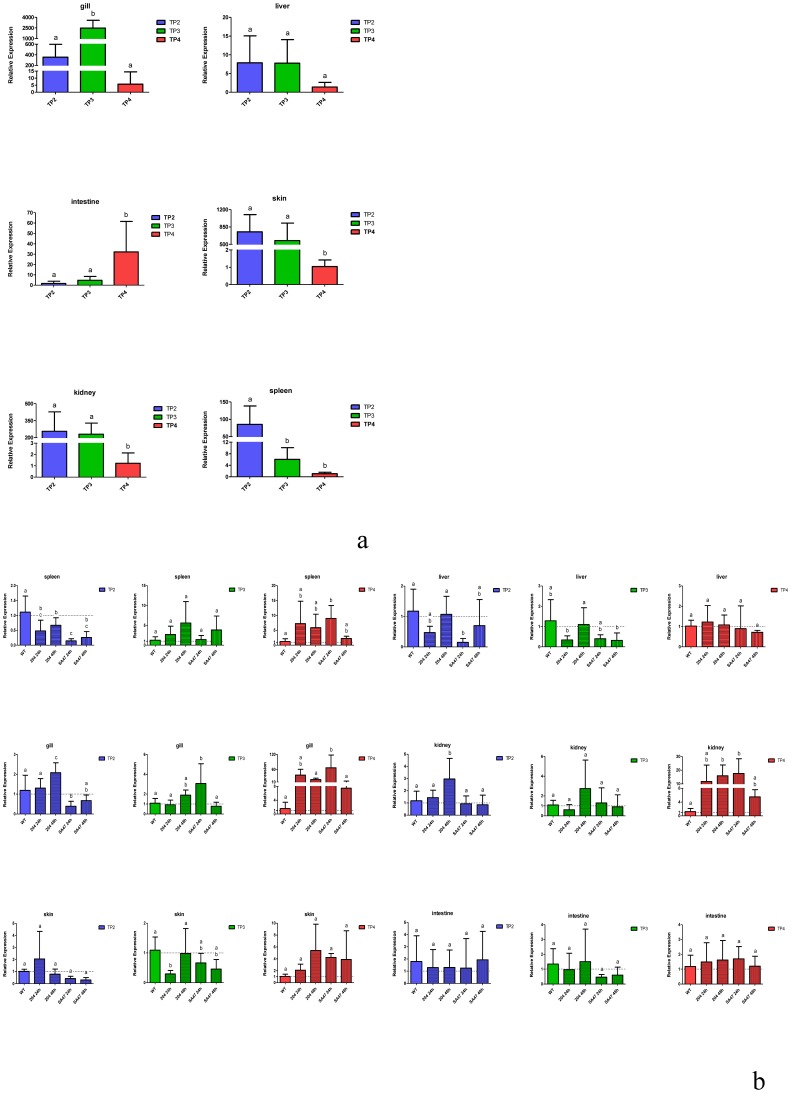
Differential expressions of Nile tilapia piscidin genes in tissues and after stimulation with bacteria. (a) A qRT-PCR analysis of Nile tilapia piscidins (TP2, -3, and -4) and ubiquitin expression in various tissues (gills, liver, intestines, skin, head kidneys (abbreviated as ‘kidneys’), and spleen) of Nile tilapia. (b) qRT-PCR analysis of Nile tilapia piscidin transcripts in various tissues (gills, liver, intestines, skin, kidney, and spleen) at two time points (24 and 48 h) after an intraperitoneal injection of *Streptococcus agalactiae* (SA47) or *Vibrio vulnificus* (204). Wild-type (WT) indicates no bacteria were injected, and the fish experienced no injection. Expression data are presented as the mean (± standard error; SE) relative quantity normalized to ubiquitin. Relative quantities were calculated to the individual (WT) with the lowest normalized piscidin expression. Data (mean ± SE) with different letters significantly differ (*p*<0.05) among tissues for single-gene comparisons.

A comparative quantitative RT-PCR analysis was used to study piscidin mRNA expression levels in different tissues, and results are presented in Fig. 4a. Each data point was normalized to ubiquitin mRNA. As shown in Fig. 4a, TP2 mRNA was most abundant in the skin, head kidneys, and spleen; TP3 mRNA was abundant in the skin, head kidneys, and gills; and TP4 mRNA was abundant in the intestines. These results suggest that except for TP4, Nile tilapia piscidins are abundantly expressed in the head kidneys and skin. However, we tried several times with different primer designs to study TP1 and TP5, but could not obtain good results, so no results are shown here for them. To study whether bacterial infection had any effects on piscidin expressions, we showed levels of Nile tilapia piscidin mRNA expressions in different tissues after *V. vulnificus* (204) stimulation (Fig. 4b) and *S. agalactiae* (SA47) stimulation (Fig. 4b) at different times (24 and 48 h). The TP2 transcript was 4∼5-fold downregulated (compared to the wild-type (WT) control, without injection) at 24 h after a *S. agalactiae* (SA47) injection in the liver, gills, and spleen. The TP2 transcript was >2-fold upregulated (compared to the WT control, without injection) 48 h after a *V. vulnificus* (204) injection in gills. The TP3 transcript was >2.5-fold upregulated (compared to the WT control, without injection) 24 h after a *S. agalactiae* (SA47) injection in gills. The TP3 transcript was <2-fold downregulated (compared to the WT control, without injection) 24 h after a *V. vulnificus* (204) injection in the skin. The TP4 transcript was >12-fold upregulated (compared to the WT control, without injection) 24 h after a *S. agalactiae* (SA47) injection and 48 h after a *V. vulnificus* (204) injection in the kidneys. The TP4 transcript was >13-fold upregulated (compared to the WT control, without injection) and 60-fold upregulated (compared to the WT control, without injection) 24 h after a *S. agalactiae* (SA47) injection in the spleen and gills.

### Antimicrobial spectrum of Nile tilapia piscidins and hemolytic activity

**Figure 5:**
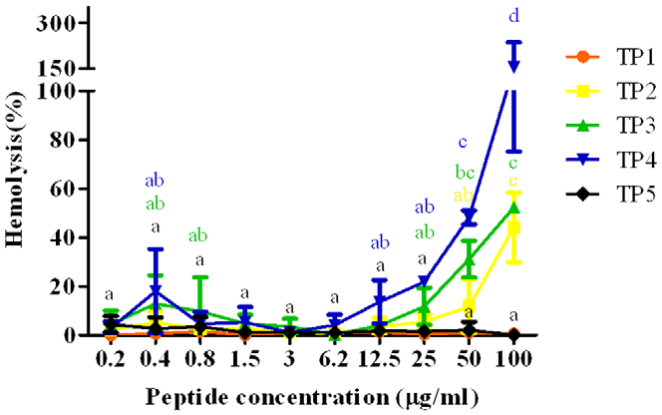
Hemolytic activity. Hemolytic activities of Nile tilapia piscidin 1∼5 (TP1, -2, -3, -4, and -5) peptides against fish erythrocytes. Data (mean ± standard error) with different letters significantly differ (*p*<0.05) among the experimental groups.

The antimicrobial spectrum was determined using the synthetic TP1∼5 peptides (Table 1; supplementary table 2). The TP3 and TP4 peptides were active against gram-positive and -negative bacteria as listed in Table 1. But TP1, -2, and -5 exhibited no antimicrobial activities against the gram-positive or -negative bacteria as listed in Table 1. TP4 presented excellent antimicrobial activity compared to cod pis 1 and grouper epinecidin-1. Furthermore, the TP1∼5 synthetic peptides were assayed for hemolytic activities in fish blood cells. The TP1 and TP5 synthetic peptides showed no hemolytic activity, and even when treated with 100 µg/ml peptide, no hemolytic activity was detected (Fig. 5). In contrast, TP4 and TP3 were hemolytic in fish red blood cells at 100 µg/ml for 100% hemolysis and 42% hemolysis, respectively (Fig. 5). TP2 achieved 60% hemolysis at 40 µg/ml.

**Table 1:** Activities of Nile tilapia piscidin 1∼5 (TP1, -2, -3, -4, and -5) peptides against gram-positive and -negative bacteria.

Bacteria Strain	Gram	TP1	TP2	TP3	TP4	TP5	cod Pis 1	Epi-1
*V. vulnificus* 204	−	>23.57	>19.52	2.44	0.03	>17.64	0.62	0.67
*A. hydrophila* BCRC 13018	−	>23.57	>19.52	>19.55	1.05	>17.64	19.78	>21.41
*V. alginolyticus*	−	>23.57	>19.52	2.44	0.03	>17.64	1.24	2.68
*P. aeruginosa* ATCC 19660	−	>23.57	>19.52	>19.55	0.52	>17.64	>19.78	10.70
*S. agalactiae* 819	+	>23.57	>19.52	9.78	2.10	>17.64	9.89	>21.41
*E. faecalis* BCRC 10066	+	>23.57	>19.52	19.55	8.39	>17.64	9.89	>21.41
*S. agalactiae* BCRC 10787	+	>23.57	>19.52	0.61	0.13	>17.64	0.31	0.33

The MIC units are in µg/ml.

### Effects of piscidins on the viability of fish cell lines

**Figure 6:**
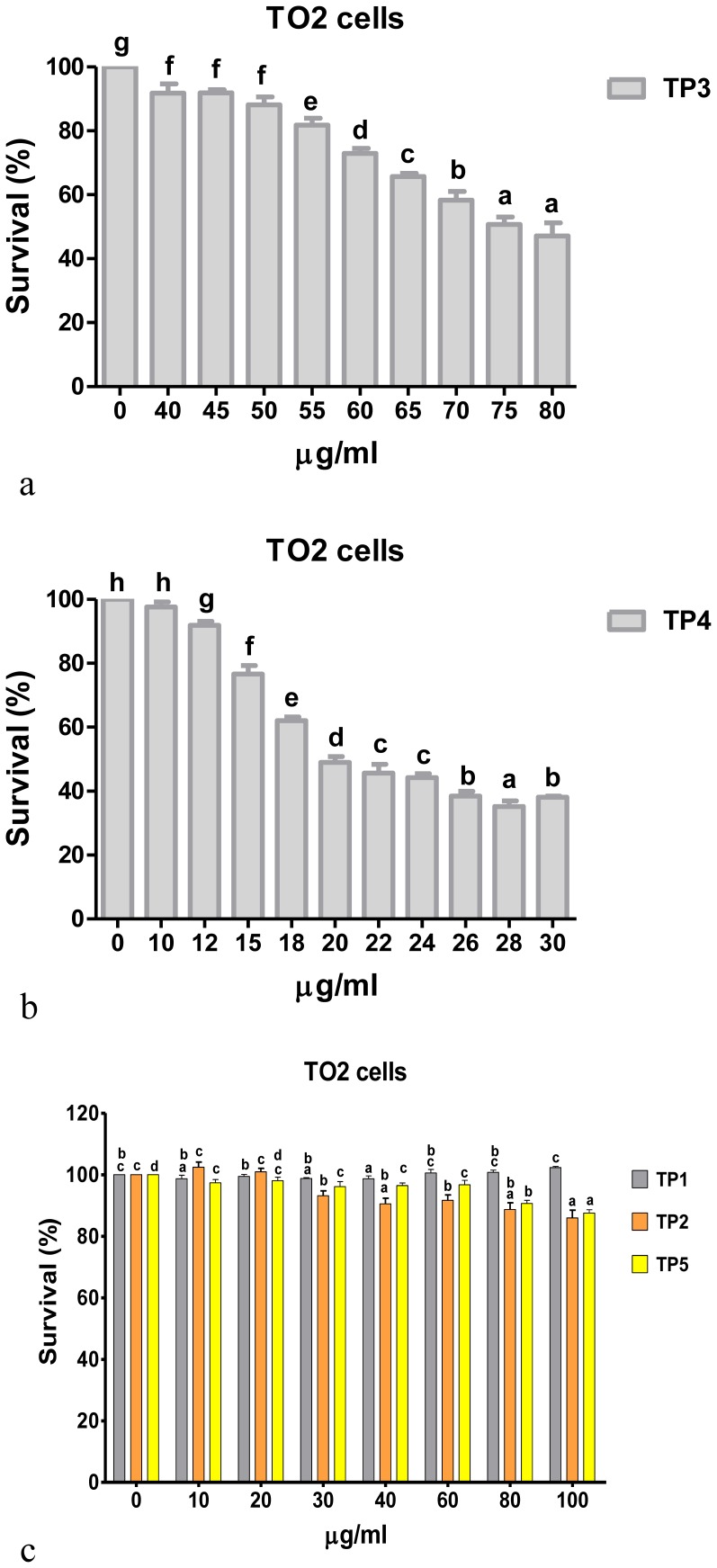
Effects of piscidins on cell proliferation. Fish cells (tilapia ovary (TO2) cell line) were treated with different doses of piscidin peptides (TP1∼5) for 24 h, followed by an MTS assay. Each concentration was repeated in eight wells for three independent experiments. Piscidin treatment affected cell viability in a dose-dependent manner. There were significant differences between treatment groups for (a) TP3 (40 µg/ml) and (b) TP4 (12 µg/ml) compared to 0 µg/ml treatment. There were significant differences between treatment groups for the lower concentrations in (c) TP2 (30 µg/ml) and TP5 (10 µg/ml) compared to 0 µg/ml treatment. But survival of cells treated with 100 µg/ml of TP1, -2, and -5 were still >80%. Data are from three separate experiments. Data (mean ± SE) with different letters significantly differ (*p*<0.05) among the experimental groups.

We examined the effects of the synthetic TP1∼5 peptides on cell viability. Treatment of tilapia ovary (TO2) cells with 0∼100 µg/ml of TP1∼5 peptides showed significantly decreased cell viability in dose-dependent manners for TP3 (Fig. 6a) and TP4 (Fig. 6b). But, TO2 cells exhibited 80% survival after treatment with the higher concentration of 100 µg/ml of TP1, -2, and -5 (Fig. 6c).

## Discussion

Five cDNAs encoding putative antimicrobial piscidin peptides from Nile tilapia, *O. niloticus*, were isolated and characterized in this study. As previously published, *Morone chrysops*×*M. saxatilis* contains two piscidin genes which are clustered on the genome [15]. In *G. morhua*, two piscidin gene were reported [35]. In this study, *O. niloticus* had five piscidins which were also clustered on the same chromosome, suggesting that piscidins may exhibit evolutionary features in fish. Multiple genes for piscidin exist in fish and may be involved in defense against bacteria in complicated aquatic environments in which fish live; these complex environments require multiple AMPs for immune protection compared to terrestrial animals. However, the first step in the cloning strategy was to search the EST database and design primers for cloning tilapia piscidin genes. The transcripts obtained were named TP1∼5, which respectively encoded putative AMPs of 68, 77, 76, 89, and 64 amino acid residues. These sequences were input for NCBI blast analyses, and results suggested that TP1 was related to *O. niloticus* pleurocidin-like peptide WF4-like (with an identity of 43%), TP2 was related to *O. niloticus* pleurocidin-like peptide WF4-like (with an identity of 100%), TP3 was related to *O. niloticus* dicentracin-like peptide (with an identity of 97%), TP4 was related to *O. niloticus* moronecidin-like peptide (with an identity of 98%), and TP5 was related to *Epinephelus bruneus* piscidin-like peptide (with an identity of 34%). Prior evidence indicated the presence of the piscidin family across a phylogenetic range of taxa; e.g., four piscidins were identified from *M. chrysops*×*M. saxatilis*. Piscidins 1, 2, and 3 are 22 amino acids long and have a highly conserved, histidine-rich, phenylalanine-rich N-terminus and a variable C-terminus [12]. Thus, the amino acid sequence homologies of Nile tilapia piscidins between Nile tilapia and Perciformes are considerably greater than those between Nile tilapia and other members of the class Osteichthyes or other vertebrate classes.

The quantitative RT-PCR analysis revealed that TP2 transcript expression was highest in the spleen and head kidneys, and lowest in the intestines. High levels of TP2 mRNA expression in the spleen and head kidneys suggest that TP2 is involved in the immune functions of these tissues in fish. Our results concurred with gaduscidin-1 and gaduscidin-2 expressions in the spleen, head kidneys, and gills of *G. morhua* [24]. It was previously reported that hybrid striped bass have high levels of moronecidin transcript expression in the head kidneys and spleen [36]. Piscidin-1 and piscidin-3 of the hybrid striped bass were reported to be localized in mast cells, while gilthead seabream piscidins are expressed in granules of circulating mast cells or in acidophilic granulocytes [12], [22]. Piscidin 3 was identified in mast cells of different organs such as the intestines and stomach, and also in uninfected fish. Piscidin 3 was also found in mast cells of gills of fish infected with ectoparasites and copepods. Piscidin 4 was found in mast cells of uninfected gills, stomach, and intestines. However, no mast cells of uninfected or parasitized intestines of *Silurus glanis* or *Barbus barbus* were positive for piscidin 3 or 4. These results suggest that piscidins 3 and 4 are encountered only in mast cells in the orders Perciformes and Gadiformes [37]. In addition, in our previous study, a tissue distribution analysis revealed high levels of *Epinephelus coioides* epinecidin-1 mRNA in the head kidneys, intestines, and skin [5]. According to those previous research results, piscidin family members were shown to be expressed in immune cells of various teleosts and to play important roles in immune-related functions in fish. Expression of the TP3 transcript in tilapia gills may be due to the gills being an important organ for fish to filter oxygen, and gills are exposed to a pathogen-filled environment and thus need to secrete AMPs to protect against invading pathogens. Gills function like the human mucosa. For example, mast cells are well-known effectors and are considered sentinels in the skin and mucosa. When a vaccinia virus infects mice, skin mast cells protect the mice against viral infection by triggering mast cell receptor S1PR2 expression and by releasing AMPs into the mucosa against pathogen infections [38]. Higher expressions of AMPs in gills and skin of sea bass suggest that these AMPs represent a first and immediate line of defense in combating pathogens and infer that the gills and skin constitute the primary physiological barriers of fish [39]. The presence of TP4 transcripts in tilapia intestines may affect the fish intestinal bacterial flora such as commensal bacteria in the intestines which engage Toll-like receptors (TLRs) and nucleotide oligomerization domain (NOD)-like receptors (NLRs) to induce specific responses by intestinal epithelial cells that produce AMPs [40]. Other AMPs, e.g., pleurocidins, are similar to piscidins and are localized in the intestines [16], [41], which indicates that they may act as a barrier to pathogen invasion at the epithelial interface. Piscidins in intestinal mast cells of gilthead seabream suggest that they possess a protective effect against microbial invasion in the intestines (and even in the gills), that are considered to be important ingress points for fish pathogens [22], [42]. Unfortunately, we did not obtain good results from our qRT-PCR analysis of TP1 and TP5. Taken together, these results support a vital role for piscidin functions that may prevent bacterial inflammatory diseases by regulating local piscidin expression.

A bacterial injection study was carried out to assess if the Nile tilapia piscidin transcripts were induced by bacterial stimulation. *Streptococcus agalactiae* (SA47) and *V. vulnificus* (204) used in this study were previously shown to strongly induce other AMP transcripts (hepcidin and cathelicidin) in the spleen and head kidneys of cod at 24 h post-infection [43]. However, the observed lack of strong upregulation of TP2 and TP3 transcripts by *S. agalactiae* (SA47) and *V. vulnificus* (204) stimulation are not unexpected, but we found significantly different expression levels of TP4 in the head kidneys, gills, and spleen of about ∼10∼60-fold after bacterial stimulation. As far as we know, AMPs are frequently constitutively expressed, such as hybrid striped bass moronecidin which was constitutively about 4-fold upregulated after *S. iniae* stimulation [36]. However, the high mRNA expression of AMP transcripts in immune-related tissues (e.g., the spleen and head kidney) suggest that TP4 may have important functions against pathogen infections. The functions of Nile tilapia piscidins and the molecular mechanisms involved in their regulation require future investigations. Furthermore, the injection method in the bacterial injection experiments needs to be discussed. An injection itself was reported to have a significant suppressive effect on piscidin expressions (particularly of cod pis 1) [25]. In that report, cod pis 1 was downregulated at the beginning of the experimental results and recovered to basal expression levels after challenge for 5 days. But respective decreases of 9.1- and 5.7-fold in the spleen and distal intestine for cod pis 1 on day 10 suggested that downregulation may be involved in the secondary innate response or cross-talk effects between the innate immune system and adaptive response [25]. In our experiments, the WT control received no injection.

Antimicrobial activities were examined by MICs. These results suggest that Nile tilapia synthesizes TP3 and TP4 peptides with antimicrobial activity, but no antimicrobial activity was determined for TP1, -2, or -5. TP4 antimicrobial activity was better than those of epinecidin-1 and cod pis 1. Antimicrobial activities of synthetic hybrid striped bass piscidins 1 and 3 were found against gram-positive and -negative bacteria at concentrations of 0.8∼25 µg/ml [12]. The fungicidal properties of synthetic hybrid striped bass piscidin 2 presented MIC values of 1.25∼6.25 µM against *Candida albicans*, *Malassezia furfur*, and *Trichosporon beigelii* [18]. Antimicrobial activities of synthetic cod piscidins 1, 2, and 2-β were found against gram-positive and -negative bacteria at concentrations of 0.04∼10 µM. Synthetic cod piscidins 1, 2, and 2-β presented a PC_min_ value of 1.25 µM against *Tetrahymena pyriformes* CBS 13-1620 [25]. It is noteworthy that TP4 was 20.66-, 41.33-, 38.03-, 4.70-, and 2.38-fold more potent than cod pis 1 in killing *V. vulnificus*, *V. alginolyticus*, *P. aeruginosa*, *S. agalactiae* 819, and *S. agalactiae* BCRC 10787. The selective antimicrobial activity of TP4 in inhibiting bacterial growth was better than that of cod pis 1, and the reasons may be related to conformational differences of the different peptides binding to host membranes [44]. The conformational differences may involve the isoelectric point (pI), net charge, and amphipathic α-helical constituents of TP4 compared to cod pis 1. One interesting research result from hybrid striped bass pis 4 showed that pis 4 possessed low helicity in membrane environments which inferred that the conformations may participate in its bioactivity [28].

Furthermore, the predicted tilapia piscidin translation products were similar to cod pis gene translation products that contain a signal peptide, a mature peptide, and a prodomain [25]. Comparison of piscidin gene sequences from different species showed some degree of similarity in piscidin signals and mature regions. Synthesis of the TP1 peptide showed four most hydrophobic residues, three net negative charges, and a theoretical acidic pI (supplementary table 2). The synthetic TP2 peptide showed one hydrophilic residue, a theoretical pI of 7.01, and potentially a linear structure (Fig. 3f). The synthesized TP3 and TP4 peptides displayed similar structural properties such as a potential amphipathic α-helical structure and cationic features. The synthesized TP5 peptide exhibited seven hydrophilic residues, and it appeared to have a similar linear structure to that of TP2. The synthesized TP1, -2, and -5 peptides presented less-potent antimicrobial activities than TP3 and TP4, and the reason may have been due to less amphipathicity and cationicity. The basis of the decreased amphipathicity and cationicity of TP5 compared to TP4 may have been the substitution of Phe for Gln at position 1, substitution of Phe for Glu at position 10, and substitution of Gln for His at position 17. Changes in the amino acids affected the antimicrobial activity due to decreased amphipathicity and cationicity, such as the example of hybrid striped bass pis 3 with a substitution of His for Gly at position 17 [12], [45]. The weak antimicrobial activities of TP1, -2, and -5 may have been due to the peptides probably adopting an amphipathic α-helical conformation without placing hydrophobic or hydrophilic residues on either side of the helix. Synthetic hybrid striped bass piscidins 1 and 2 showed stronger hemolysis than piscidin 3, and piscidin 3 had relatively weak antibacterial activity [12]. Comparison of our results (TP3 and TP4) with hybrid striped bass piscidins 1 and 2 showed that similar hemolytic activities existed for TP3 and TP4. Therefore, differences in the biological functions and the structural diversity of piscidin peptides found in tilapia may be because tilapia have to accommodate to new surroundings. Specific positive selection sites in the mature region of pis 1 with a substitution cause variations in the amphipathicity and hydrophobicity and affect its biological functions [23], [25].

In a previous study, antimicrobial activities of piscidins were demonstrated against gram-positive and -negative bacteria. The results presented in this study showed that TP3 and TP4 can rapidly initiate cytotoxicity and significantly inhibited the proliferation of fish cells in a dose-dependent manner. TP1 and TP5 showed no cytotoxicity toward fish blood cells after treatment with 100 µg/ml peptide concentrations. Comparison of cytotoxic effects in mammalian cells to those of HeLa cells indicated that HeLa cells were significantly more sensitive than other tested cell lines when treated with 40 µg/ml TP3, which produced a 20% survival rate (Supplementary Fig. 4a). Meanwhile, proliferation was relatively unaffected by TP3 treatment at 40 µg/ml in non-tumorigenic MRC-5 cells (80% survival) (Supplementary Fig. 4a). Additionally, TP3 also showed low hemolytic activity toward human erythrocytes compared to TP4 (Supplementary Fig. 4b). These supplementary experimental results indicate that TP3 is relatively nontoxic to cells which are not associated with tumors, but is especially active toward human HeLa cervical cells. The mechanisms for the selective killing of tumor cells by TP3 are unclear. TP2 is hemolytic but not antibacterial, which suggests that its functions differ from those of the other peptides. The mechanisms for the selective hemolytic but not antibacterial property of TP2 are unclear. The hemolytic effect of TP4 began at 0.4∼0.8 µg/ml in fish blood cells and human blood cells, concentrations that correspond to the MIC value. In addition, there was no different cytotoxicity of TP4 between normal and cancer cells or fish cells. These two observations are relevant and point out the absence of selectivity of TP4. Although we found no selective killing of tumor cells by TP1, -2, or -5, we also could not find a significant inhibitory effect on proliferation of TO2 cells (fish cells). These peptides may play roles in other immune-related functions in fish. From the results of the hemolytic activity against fish blood cells and the effects of piscidins on fish cell proliferation mentioned above, it was interesting to ascertain the hemolytic effects and cell proliferation of TP1∼5 in mammalian erythrocytes and cell lines. Therefore, our data presented in Supplementary Fig. 4 show conserved biological functions for TP1∼5 in fish and mammalian cells. This can be ascribed to the composition of cell membranes and the dispersion of phospholipids, which can cause cell selectivity and susceptibility of cancer cells to lysis. Variability in the cytotoxicity of tilapia piscidin peptides was observed when they were tested against different types of cancer cells. Results suggest that the different sensitivities may be attributed to differences in the cell membrane composition, fluidity, and piscidin hydrophobic properties [18], [46], [47]. Higher hydrophobicity was correlated with stronger hemolytic activity in α-helical AMPs and indicated a greater ability to kill cancer cells.

In conclusion, we analyzed gene expression changes of five Nile tilapia (*O. niloticus*) piscidins during *V. vulnificus* (204) and *S. agalactiae* (SA47) stimulation. Inferring their functions through these results showed that Nile tilapia piscidin expression levels responded to bacterial infections. Following a predictive structural analysis, the N-terminal end structures of Nile tilapia piscidins were found to be conserved. From a qRT-PCR analysis, we determined that TP2 mRNA was most abundant in the skin, head kidneys, liver, and spleen; TP3 mRNA was abundant in the gills; and TP4 mRNA was abundant in the intestines. However, MICs demonstrated that two of the Nile tilapia piscidins had antibacterial ability, hemolytic activity, and antitumor cell activity. Furthermore, identifying receptors for the five types of Nile tilapia piscidins will facilitate our understanding of fish innate immunity. Also utilizing piscidin peptides to develop peptide drugs could improve resistance to pathogenic infections, which is important for the success and growth of the aquaculture industry and for human health.

## Supporting Information

Figure S1 **Standard curves for determining PCR efficiencies.** The slope (m) was defined from the formula Ct = m (log Q)+c, where Ct is the threshold cycle, Q is the initial amount of cDNA, and c is the intercept on the ordinate axis. UBC, ubiquitin gene.
(TIF)

Figure S2 **Boxplots of ubiquitin gene (reference gene) Ct distributions among the gills, liver, intestines, skin, head kidneys (abbreviated as kidneys), and spleen throughout the study.** Boxes are interquartile ranges, bars in boxes are medians, and lines extending from the boxes are minimal and maximal values. UBC, ubiquitin gene.
(TIF)

Figure S3 **Nucleotide (nt) sequence of Nile tilapia (** ***Oreochromis niloticus*** **) piscidin (TP1∼5) sequences and the predicted amino acid (aa) sequence of the gene.** Nucleotides are numbered beginning with the first nucleotide at the 5′ end. An asterisk (*) indicates a stop codon.
(TIFF)

Figure S4 **In vitro assays used HeLa (ATCC:** CCL-2™**), RAW264.7 (ATCC:** TIB-71™**), and MRC-5 (ATCC:** CCL-171™**) cells.** Tumor cells (HeLa and RAW264.7 cells) and normal cells (MRC-5) were treated with different doses of piscidin peptides (TP1∼5) for 24 h, followed by an MTS assay. Each concentration was repeated in eight wells for three independent experiments. Results are expressed as a percentage of the survival rate of viable cells. Piscidin treatment affected cell viability in a dose-dependent manner. There were significant differences between treatment groups for TP4 and TP3. Data are from three separate experiments. Hemolytic activities of TP1∼5 against human erythrocytes. The experimental methods for the hemolytic activity analysis are described in “Materials and methods”. This hemolytic assay was performed in accordance with the *Declaration of Helsinki* as a statement of ethical principles. Healthy human erythrocytes (2×10^7^ cells) were used in the hemolytic assay. The hemolysis assay was performed in triplicate. Cell proliferation results for TP1∼5 are shown in Supplementary Figure 4a. Hemolytic activities of TP1∼5 against human erythrocytes are shown in Supplementary Figure 4b.
(TIFF)

Table S1 Primer amplification efficiencies and amplicon sizes (bp).
(DOC)

Table S2 Information on TP1∼5 coding region sequences and the synthesized peptide sequences indicated in red color used in this manuscript. The compute theoretical isoelectric point (pI) and molecular weight (Mw) of the piscidin amino acid sequences were input to the website (http://web.expasy.org/compute_pi/) for analysis.
(DOC)

Table S3 Species, accession numbers, and gene names for the sequences alignment analysis in Fig. 2.
(DOC)
