# Supplementary figures and images for: Five Different Piscidins from Nile Tilapia, Oreochromis niloticus: Analysis of Their Expressions and Biological Functions
Source: PLoS One. 2012 Nov 30;7(11):e50263. doi: 10.1371/journal.pone.0050263 (PMC3511469; doi:10.1371/journal.pone.0050263)

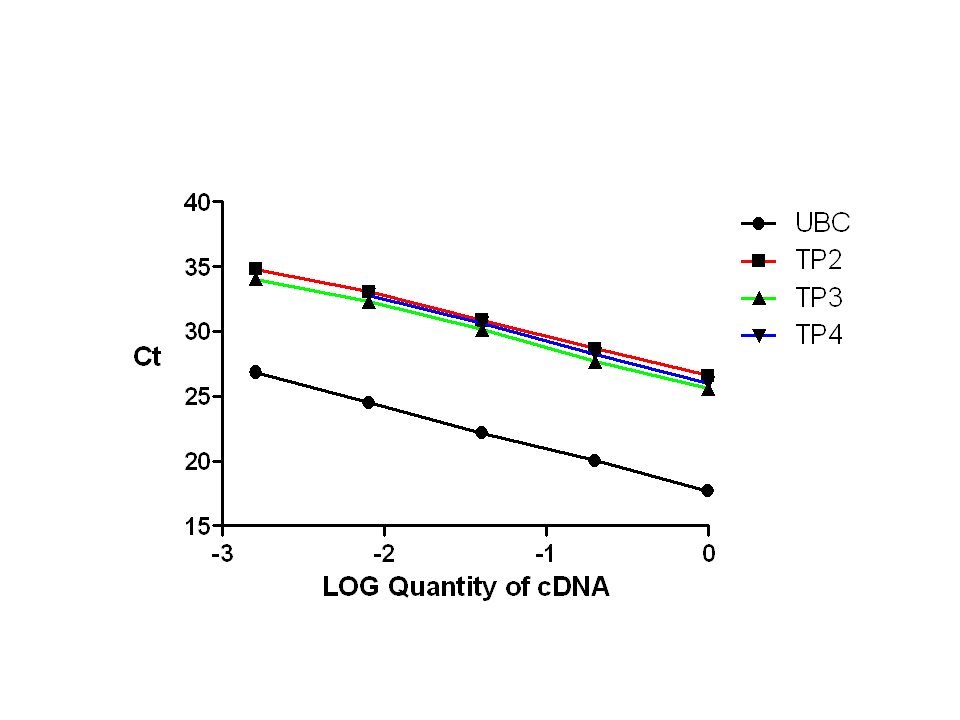

Supplement: Figure S1 — Standard curves for determining PCR efficiencies. The slope (m) was defined from the formula Ct = m (log Q)+c, where Ct is the threshold cycle, Q is the initial amount of cDNA, and c is the intercept on the ordinate axis. UBC, ubiquitin gene. (TIF) [file pone.0050263.s001.tif]
