# Supplementary material for: Five Different Piscidins from Nile Tilapia, Oreochromis niloticus: Analysis of Their Expressions and Biological Functions
Source: PLoS One. 2012 Nov 30;7(11):e50263. doi: 10.1371/journal.pone.0050263 (PMC3511469; doi:10.1371/journal.pone.0050263)
Supplement: Table S1 — Primer amplification efficiencies and amplicon sizes (bp). (DOC) [file pone.0050263.s005.doc]

Supplementary table 1

Primer amplication efficiencies# and amplicon size (bp)

# Primer amplification efficiencies (E) were calculated from the slope values of the standard curves according to the equation: E=10(1/-slope)-1.
