# Supplementary material for: Five Different Piscidins from Nile Tilapia, Oreochromis niloticus: Analysis of Their Expressions and Biological Functions
Source: PLoS One. 2012 Nov 30;7(11):e50263. doi: 10.1371/journal.pone.0050263 (PMC3511469; doi:10.1371/journal.pone.0050263)
Supplement: Table S2 — Information on TP1∼5 coding region sequences and the synthesized peptide sequences indicated in red color used in this manuscript. The compute theoretical isoelectric point (pI) and molecular weight (Mw) of the piscidin amino acid sequences were input to the website (http://web.expasy.org/compute_pi/) for analysis. (DOC) [file pone.0050263.s006.doc]

Supplementary table 2.

| Gene name | Amino acid sequences | Theoretical pI (isoelectric point) and Mw (molecular weight) |
| --- | --- | --- |
| TP1 coding region | MKSAVIFLALFMVFMMAEPGECFDWDSVLKGVEGFVRGYFGKEKAKELVKSLKADFQNYKHLRQREFD | 7.86 / 7958.33 |
| TP1 synthesis peptide used in this study | FDWDSVLKGVEGFVRGYF | 4.56 / 2121.38 |
| TP2 coding region | MKCAAVFLMLSMVILMAEPGECIWDAIFHGAKHFLHRLVNPGGKDAVKDVQQKQEQQKDQELDKRAISYHPRRLNFD | 7.84 / 8893.36 |
| TP2 synthesis peptide used in this study | GECIWDAIFHGAKHFLHRLVNP | 7.01 / 2560.96 |
| TP3 coding region | MKCTMLFLVLSMVVLMAEPGEAFIHHIIGGLFSVGKHIHSLIHGHGNVKQQQQQQEQLNQRSFNREQFKRERAAFN | 9.62 / 8771.18 |
| TP3 synthesis peptide used in this study | FIHHIIGGLFSVGKHIHSLIHGH | 8.79 / 2557.00 |
| TP4 coding region | MKCTILFLVLSMVVLMAEPGEGFIHHIIGGLFSAGKAIHRLIRRRRRGELQLEQELQQQLEQLEKLQQQEKLNQRFNREQLKRERVAFN | 10.15 / 10566.41 |
| TP4 synthesis peptide used in this study | FIHHIIGGLFSAGKAIHRLIRRRRR | 12.70 / 2981.60 |
| TP5 coding region | MKSAIIFLVFTLVLFMADPAELQLQGKQVSGEVVQKVLQELIQSVAKPGPKDVNFNEFEDAVMS | 4.56 / 7067.29 |
| TP5 synthesis peptide used in this study | QLQGKQVSGEVVQKVLQELIQSVAKP | 8.50 / 2834.31 |
