# Supplementary material for: Five Different Piscidins from Nile Tilapia, Oreochromis niloticus: Analysis of Their Expressions and Biological Functions
Source: PLoS One. 2012 Nov 30;7(11):e50263. doi: 10.1371/journal.pone.0050263 (PMC3511469; doi:10.1371/journal.pone.0050263)
Supplement: Table S3 — Species, accession numbers, and gene names for the sequences alignment analysis in Fig. 2. (DOC) [file pone.0050263.s007.doc]

| **Scientific names** | **Common names** | **Genes** | **ACCESSION NO.** |  |
| --- | --- | --- | --- | --- |
| *Anoplopoma fimbria* | Sablefish | moronecidin precursor | BT082221 |  |
|  |  | dicentracin precursor | BT082403 |  |
| *Dicentrarchus labrax* | European seabass | dicentracine | AY303949 |  |
| *Epinephelus akaara* | Hong Kong grouper | piscidin-like peptide | EU741828 |  |
| *Epinephelus bleekeri* | Duskytail grouper | piscidin-like antimicrobial peptide precursor | HQ437912 |  |
| *Epinephelus bruneus* | Longtooth grouper | piscidin-like peptide | JN216987 |  |
| *Epinephelus coioides* | Orange-spotted grouper | piscidin-like peptide | EU741829 |  |
|  |  | piscidin-like antimicrobial peptide precursor | HQ437913 |  |
| *Epinephelus fuscoguttatus* | Brown-marbled grouper | piscidin-like peptide | GU592793 |  |
| *Epinephelus malabaricus* | Malabar grouper | piscidin-like antimicrobial peptide precursor | HQ437914 |  |
| *Gadus morhua* | Atlantic cod | gaduscidin-1 | HM015527 |  |
|  |  | gaduscidin-2 | HM015528 |  |
| *Glyptocephalus cynoglossus* | Witch | pleurocidin-like peptide GcSc4C5 | AY273176 |  |
|  |  | pleurocidin-like peptide GcSc4B7 | AY273177 |  |
|  |  | pleurocidin-like peptide GC3.8 | AY273178 |  |
|  |  | pleurocidin-like peptide GC3.2 | AY273179 |  |
| *Haplochromis burtoni* | Burton's haplo | EST clone | DY628409 |  |
| *Hippocampus kuda* | Spotted seahorse | antimicrobial protein plp | AY864343 |  |
|  |  | ASABF-like antimicrobial protein | EU556318 |  |
| *Hippoglossoides platessoides* | American plaice | pleurocidin-like peptide AP1 (ple) | AY273172 |  |
|  |  | pleurocidin-like peptide AP2 (ple) | AY273173 |  |
|  |  | pleurocidin-like peptide AP3 (ple) | AY273174 |  |
| *Hippoglossus hippoglossus* | Atlantic halibut | pleurocidin-like peptide Hb26 | AY273180 |  |
|  |  | pleurocidin-like peptide Hb18 | AY273181 |  |
| *Larimichthys crocea* | Large yellow croaker | piscidin-like peptide | EU741827 |  |
| *Limanda ferruginea* | Yellowtail flounder | pleurocidin-like peptide YT2 | AY273175 |  |
| *Limanda limanda* | Common dab | pleurocidin prepropolypeptide | DQ248966 |  |
| *Morone chrysops* | White bass | moronecidin prepropeptide | AF332621 |  |
| *Morone chrysops x*  *Morone saxatilis* | Hybrid striped bass | piscidin-4 precursor | HM596029 |  |
|  |  | piscidin-5 precursor | HM596030 |  |
| *Morone saxatilis* | Striped bass | moronecidin precursor | AF385583 |  |
| *Pseudopleuronectes americanus* | Winter flounder | pleurocidin-like peptide WFYT | AAQ16622 |  |
|  |  | pleurocidin-like peptide WFX | AAQ16623 |  |
|  |  | pleurocidin-like peptide WF2 | AAQ16625 |  |
|  |  | pleurocidin | AF210242 |  |
|  |  | pleurocidin-like prepropolypeptide (ple1) | AF301506 |  |
|  |  | pleurocidin-like prepropolypeptide (ple3) | AF301508 |  |
|  |  | pleurocidin-like prepropolypeptide (ple1a) | AF301509 |  |
|  |  | pleurocidin prepropolypeptide (ple4) | AF301515 |  |
| *Siniperca chuatsi* | Mandarin fish | moronecidin | AY647433 |  |
